# Disseminated and late metastatic disease from nasal pit leiomyosarcoma after radical surgical resection. Case report of a singular presentation of a rare disease

**DOI:** 10.1186/s13569-017-0078-2

**Published:** 2017-06-06

**Authors:** Enrico Pinotti, Marta Sandini, Simone Famularo, Marta Jaconi, Fabrizio Romano, Luca Nespoli, Luca Gianotti

**Affiliations:** 1 Department of Surgery, School of Medicine and Surgery, San Gerardo Hospital (4° Piano A), University of Milano Bicocca, Via Pergolesi 33, 20900 Monza, Italy; 2 Unit of Pathology, School of Medicine and Surgery, San Gerardo Hospital, University of Milano Bicocca, Monza, Italy

**Keywords:** Leiomyosarcoma, Nasal pit, Metastasis, Follow up, Pancreas

## Abstract

**Background:**

Leiomyosarcoma of the head and neck is a rare cancer with high local aggressiveness. Radical surgery and adjuvant treatment offer the best chance for cure, nonetheless 5-years recurrence rate remains high. Despite international guidelines are available for soft tissue sarcomas, no recommendations are specifically endorsed for leiomyosarcoma of the head and neck, due to the rarity of its presentation and consequently scarcity of data on long-term outcome.

**Case presentation:**

A 50-year old woman, operated 10 years before for leiomyosarcoma of the nasal pit and with negative 5-years follow-up, was admitted to our ward for impairment of the hepatic function. Total-body CT scan detected multiple localizations at lungs, kidneys, pancreas, bones, muscles, lymph nodes and thyroid. The pathologic report after lung biopsy confirmed the diagnosis of metastasis from leiomyosarcoma and the patients was scheduled for first line chemo-radiotherapy.

**Conclusions:**

Despite adequate primary treatment, distant and disseminated metastatic disease may be not excluded in leiomyosarcoma of the head and neck.

## Background

Soft tissue sarcomas (STS) are malignant tumors of mesenchymal origin, arising in connective tissue and extra-osseous connective tissue and representing about 1% of all adult cancers [1]. A rare histotype is represented by leiomyosarcoma of the head and neck, which accounts less than 0.1% of the tumors of this anatomic region [2]. When arisen in the area of head and neck its typical presentation involves the oral cavity, scalp, paranasal sinuses and jaws [3].

Despite international guidelines are available for staging work-up and therapeutic option of STS [4, 5], due to its uncommon occurrence, leiomyosarcoma of the head is burden by scarcity of data concerning long-term outcome and optimal management. Radical surgical resection associated with adjuvant chemoradiation seems to offer the best chance for cure [6].

Accurate data on long-term outcome are lacking. The largest retrospective series on 130 patients with sarcomas of the head and neck reported a 68% 5-years metastasis free-survival, a local recurrence rate within 5 years of more than 50%. In nearly half of metastatic patients it is observed a concomitant relapse in the primary site, suggesting high local aggressiveness. The classical sites of distant dissemination are liver, bone, brain and skin [7].

This article presents a case of a 50-year old woman with disseminated metachronous metastases of leiomyosarcoma diagnosed 10 years after being operated and treated with radiotherapy for a primary tumor of the nasal pit and with a negative 5-years follow-up.

## Case presentation

A 50-year old woman suffering from psoriasis underwent a radical resection of a leiomyosarcoma of the nasal pit in the year 2006 she was subsequently treated with 13.2 Gy/12 fractions of radiotherapy. In the year 2011 she completed a 5-years negative follow up. No cases of soft tissue sarcoma were noticed among her relatives.

In April 2016 the patient was admitted to our surgical ward for the incidental finding of abnormal values of hepatic function laboratory tests: alkaline phosphatase 286 U/L; gamma-glutamyl transferase 621 U/L; bilirubin 0.8 mg/dL; AST 488 U/L; ALT 1248 U/L. Patient history revealed abdominal discomfort in the previous 4 months. For this reason she underwent an abdominal ultrasound that excluded pathological findings. She had no symptoms or signs of pancreatic exocrine or endocrine insufficiency and no weight loss.

**Fig. 1:**
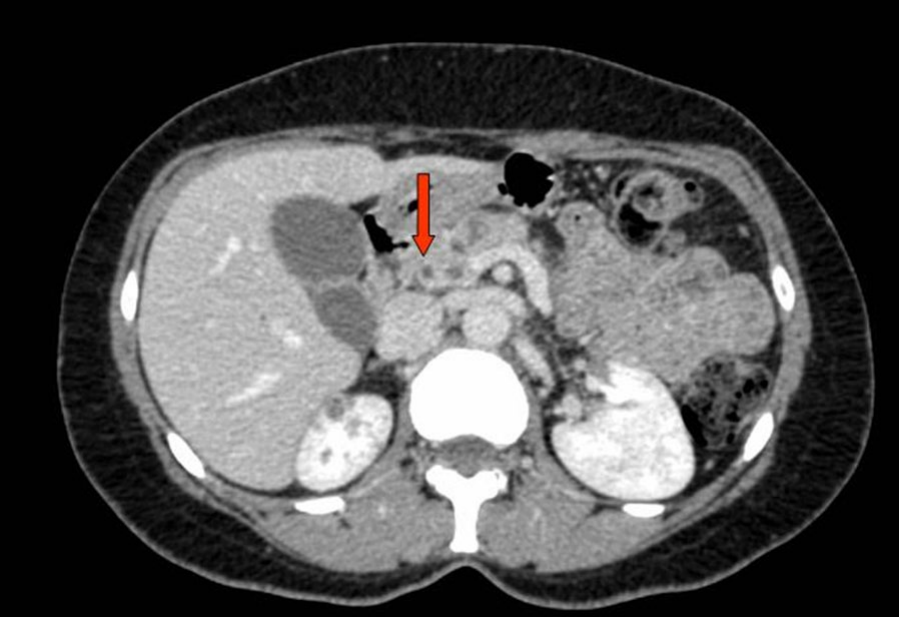
Contrast-enhanced CT-scan with detection of multiple hypodense pancreatic lesions

**Fig. 2:**
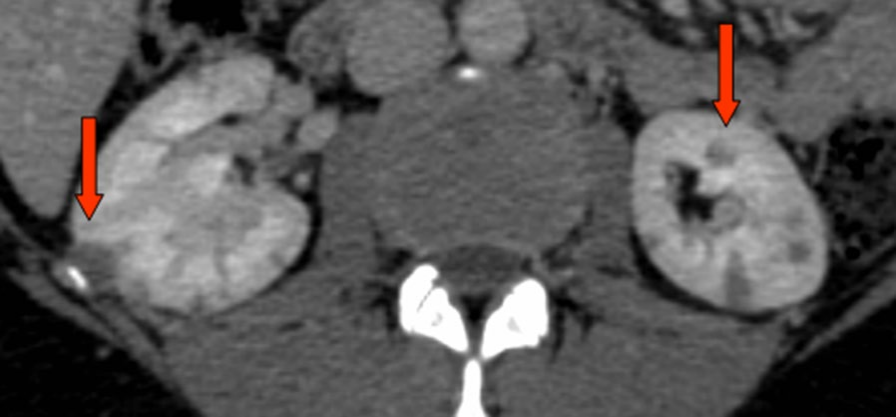
Contrast-enhanced CT-scan with detection of multiple, hypodense and bilateral kidney lesions

**Fig. 3:**
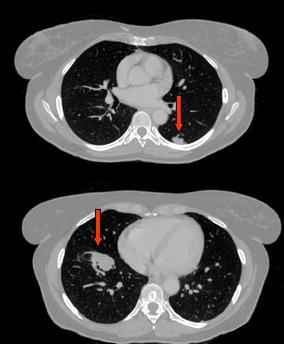
Bilateral lung metastases at CT-scan

After admission, the patient repeated an abdominal ultrasound scan that showed dilation of the intrahepatic bile ducts and a solid hypoechogenic 4 cm mass of the head of the pancreas. She was then submitted to a total-body contrast-enhanced TC-scan with detection of multiple hypodense pancreatic lesions (Fig. 1), bilateral hypodense renal lesions (Fig. 2) and bilateral lung lesions (Fig. 3). Liver and kidney lesions appear hypodense both in venous phase and after contrast enhancement. No malignancy was detected in the brain at CT-scan. The radiologic report was suggestive for primary lung cancer with multiple abdominal metastases. Thus, the patient received a CT-guided core needle lung biopsy for histological characterization.

**Fig. 4:**
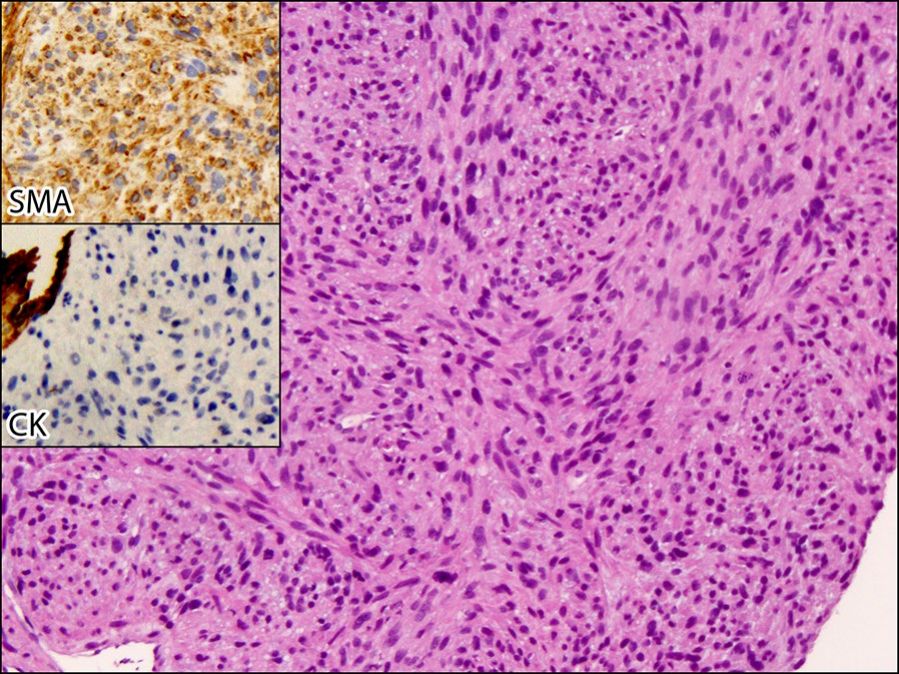
Histologic features of the core-needle biopsy. The lesion was constituted of malignant smooth muscle fascicles, as demonstrated by the immunohistochemistry for smooth muscle actin (SMA) and pool cytokeratin (CK, which is shown to have a positive control on the normal lung parenchyma *left*)

The biopsy (Fig. 4) was constituted of lung parenchyma invaded by a population of fusiform elements with marked nuclear pleomorphism, hyperchromasia and high mitotic activity growing in a fascicular pattern. Immunohistochemistry of the neoplastic elements revealed them to be positive for smooth muscle actin, muscle-specific actin and desmin, and negative for pool cytokeratin markers (AE1–AE3). The immunophenotype of the lesion was thus compatible with metastases from the previous leiomyosarcoma. The histological slides were reviewed in a sarcoma referral center by an expert pathologist, who confirmed the diagnosis.

To further explore the maxillary region, a magnetic resonance was prescribed with the evidence of an additional lesion at left supraclavicular lymph nodal region. We completed the oncological staging by a total-body PET scan, which confirmed the multiple metastases at lungs, kidneys and pancreas, multiple high-metabolic intensity signals at bones (left occipital condyle, ribs, vertebrae, and pelvis), muscles (right pectoralis major, left vastus intermedius and left rhomboid) thoracic lymph nodes, and thyroid gland.

The patient was evaluated for oncological treatment at a referral center. She started radiochemotherapy with 6 cycles of anthracycline and dacarbazine, plus single-fraction 8 Gy on selected bone localization. At restaging with total-body enhanced CT-scan and total-body PET-CT scan, partial remission of the disease was observed, according to RECIST criteria [8]. At last clinical follow-up at our Institution (November 2016), the patient was still undergoing single-drug chemotherapy scheme with systemic dacarbazine, showing good quality of life.

## Conclusions

Leiomyosarcoma of the head is a very uncommon malignant disease, characterized by high local aggressiveness. Since the incidence of this type of neoplasm is low, all patients should be treated by multidisciplinary team at referral centers.

It has been suggested that radical surgical resection followed by adjuvant chemoradiation or neoadjuvant local radiation with postoperative chemotherapy, may represent the best opportunities for cure, but so far no gold standard treatments and follow-up protocols have been clearly recommended for head and neck leiomyosarcoma [9].

Our patient presented with disseminated metastatic disease. We initially hypothesized that it might origin by a new growth carcinoma. The pathologic report was edited after comparing the lung biopsy with the surgical specimen of the former leiomyosarcoma and in both cases similar morphologic and immunohistochemistry patterns were found. Despite the resection of the former leiomyosarcoma dated 10 years negative follow up, the rarity of the disease and its pathognomonic features at pathology suggested that a relapse of the previous malignancy might be more likely than a new primary tumor.

Metastases from leiomyosarcoma have been commonly described following local recurrence and, in case of primary lesion at head and neck, distant relapse is usually detected in the cervical region or in the lungs [10], while no cases of abdominal metastases have been reported. Beside pulmonary metastases (a common feature of leiomyosarcoma), the patient presented with multiple abdominal metastases in the kidneys and in the pancreas. Both the latter presentations are peculiar because distant localization in the kidney is anecdotal [11] and pancreatic metastases are often solitary and found within a short period of time.

Suh et al. [12] recently reviewed their experience on 13 cases of pancreatic metastases from leiomyosarcoma. The majority of patients had the primary tumor originating from uterus, retroperitoneum or lower extremities and in most of cases a single metastasis was described with a median resection-to-relapse time of 17 months (range 1–77 months). Multiple site metastases or synchronous spread presentation of leiomyosarcoma have been observed, but only in patients with impaired immune function due to radiation therapy, HIV, other neoplasms or in transplant receipts. In those cases, Epstein–Barr virus infection seems to play a key-role in the pathogenesis of the disease [13, 14]. Since our patient did not fulfill the above conditions, a reliable reason for multiple tumor localization remains uncertain. Hematogenous dissemination is a common trait of leiomyosarcoma [8]. Thus, it may be hypothesized that the abdominal presentation may have followed the lung metastasis process, through systemic blood flow, but we did not observe hepatic localization. This remark seems peculiar, if considering the common origin of the pancreatic and liver arterial blood flow from the celiac trunk. The present case was characterized by the late and extensive appearance of the metastatic disease, absence of local recurrence, young age and lack of concomitant immune impairment, suggesting a very high aggressive biologic behavior of the tumor.

Local recurrences have been described up to 15 years after primary resection [7]. Similarly to our observation, Minni et al. reported that three patients, out of a series of four, developed metachronous pancreatic metastases at an interval of 9.2 years after the diagnosis of primary tumor [15]. Nonetheless relapse appears more often in the first 3 years after surgery [16] and recurrence after 5 years remains an uncommon manifestation [17].

In conclusion, despite radical resection, adjuvant chemoradiation and accurate long-term follow-up, distant and disseminated recurrence of the disease may not be excluded in leiomyosarcoma of the head and neck. Since prompt multimodal treatment of metastatic disease may improve overall survival, the suggested 10-years follow-up should be fulfilled. The adequate timing to interrupt surveillance after resection and its cost-effectiveness need further investigation.
